# Predicting the risk of mortality and rehospitalization in heart failure patients: A retrospective cohort study by machine learning approach

**DOI:** 10.1002/clc.24280

**Published:** 2024-05-20

**Authors:** Reza Tabrizi, Marzieh Ketabi, Aref Andishgar, Mohebat Vali, Zhila Fereidouni, Maryam Mojarrad Sani, Ashkan Abdollahi, Abdulhakim Alkamel

**Affiliations:** ^1^ Noncommunicable Diseases Research Center Fasa University of Medical Sciences Fasa Iran; ^2^ Clinical Research Development Unit, Valiasr Hospital Fasa University of Medical Sciences Fasa Iran; ^3^ Student Research Committee Fasa University of Medical Sciences Fasa Iran; ^4^ USERN Office Fasa University of Medical Sciences Fasa Iran; ^5^ Student Research Committee Shiraz University of Medical Sciences Shiraz Iran; ^6^ Department of Medical Surgical Nursing Fasa University of Medical Sciences Fasa Fars Iran; ^7^ School of Medicine Tehran University of Medical Sciences Tehran Iran; ^8^ School of Medicine Shiraz University of Medical Sciences Shiraz Iran

We welcome and appreciate the comments raised by Nabi et al. related to our recent publication “Predicting the risk of mortality and rehospitalization in heart failure patients: A retrospective cohort study by machine learning approach.”^1^ In this article, we used machine learning algorithms to predict mortality and readmission among heart failure patients has the potential to significantly improve patient care and outcomes. Our emphasis on the importance of early detection of high‐risk heart failure patients through machine learning models aligns with the current trend toward personalized and patient‐centered healthcare. By using predictive analytics, clinicians can identify individuals at risk of adverse events and provide tailored interventions, ultimately leading to better patient outcomes.

In response to the comments raised in this letter, we present a few clarifications here. The study evaluated the performance of various machine learning algorithms in predicting heart failure outcomes and provided valuable insights into the potential of AI in healthcare. The identification of important predictors such as length of hospital stay, hemoglobin levels, and family history of MI highlights the significance of these factors in predicting readmission and mortality among heart failure patients.

As we have previously mentioned, you also discussed the limitations of the study, including the necessity for prospective studies to address potential selection bias and broader geographical studies to enhance the generalizability of the findings.^2^ These aspects highlight the bias that is inherent in observational studies and is not within our control.

In conclusion, integrating machine learning to predict heart failure outcomes offers a promising way to enhance patient care, optimize resource allocation, and reduce healthcare costs. Continued research and implementation of AI‐based predictive models in various geographical locations have the potential to revolutionize the management of heart failure and improve patient outcomes globally.

Future studies should be conducted prospectively to confirm the generalizability of these machine learning algorithms. Additionally, other advanced artificial intelligence models can be used in future studies that can improve the management of heart failure patients.

## References

[1] Ketabi M , Andishgar A , Fereidouni Z , et al. Predicting the risk of mortality and rehospitalization in heart failure patients: a retrospective cohort study by machine learning approach. Clin Cardiol. 2024;47(2):e24239. 38402566 10.1002/clc.24239 PMC10894620

[2] Angraal S , Mortazavi BJ , Gupta A , et al. Machine learning prediction of mortality and hospitalization in heart failure with preserved ejection fraction. JACC: Heart Failure. 2020;8(1):12‐21. 31606361 10.1016/j.jchf.2019.06.013

